# Geographic Distribution and Temporal Trends of Zika Virus Outbreaks in India (2016–2023): Insights From the Integrated Disease Surveillance Programme Data

**DOI:** 10.7759/cureus.67068

**Published:** 2024-08-17

**Authors:** Dinesh Asokan, Siva Prasad Reddy Bommu, Anjali Mall, Geeta Pardeshi

**Affiliations:** 1 Community Medicine, Grant Government Medical College and Sir JJ Group of Hospitals, Mumbai, IND

**Keywords:** geographic information system, integrated disease surveillance programme, public health, seasonal trends, gis, india, zika virus

## Abstract

Introduction

This study examines the geographic distribution and temporal trends of Zika virus (ZIKV) outbreaks in India from 2016 to 2023 using data from the Integrated Disease Surveillance Programme (IDSP). The burden of ZIKV in India has risen due to its rapid spread and significant health impacts. Existing literature highlights seasonal and geographic patterns but lacks a comprehensive, long-term analysis specific to India. This study addresses this gap by analyzing trends over seven years to inform better public health responses.

Methods

A secondary data analysis was conducted using publicly available data from the IDSP on reported Zika cases from January 2016 to December 2023. Descriptive statistical methods and geographic information system (GIS) mapping techniques were employed to analyze the geographic distribution and temporal trends of ZIKV outbreaks in India. The data were analyzed and visualized using R software version 4.3.2 (R Foundation for Statistical Computing, Vienna, Austria), with heat maps and choropleth maps to identify hotspots, and line diagrams to identify temporal trends.

Results

Zika outbreaks predominantly occurred during the post-monsoon season, accounting for 47.62% (n = 10) of the total 21 outbreaks, followed by the monsoon season with 33.33% (n = 7), and summer with 19.05% (n = 4). Two deaths were reported during a significant outbreak in Madhya Pradesh in 2018. Temporal trends indicated notable spikes in cases in 2018 (131 cases) and 2021 (234 cases), with no cases reported in 2019 and 2020. The geographic distribution maps highlighted significant concentrations of ZIKV outbreaks in specific districts within Uttar Pradesh, Madhya Pradesh, and Kerala.

Discussion

The study identified seasonal patterns, with most cases occurring in the post-monsoon season. The geographic spread of the ZIKV was observed in eight states from 2016 to 2023. GIS identified three hotspots in Uttar Pradesh, Madhya Pradesh, and Kerala.

Conclusion

The study highlights the need for heightened surveillance and targeted intervention preparedness during high-risk seasons. Enhancing testing facilities and data reporting systems could improve outbreak identification, management, and response.

## Introduction

The Zika virus (ZIKV) has recently reemerged as a significant public health threat in Maharashtra and across India. In response to reported cases of Zika virus in Maharashtra, the Director General of Health Services (DGHS) at the Ministry of Health and Family Welfare has issued a comprehensive advisory to all states emphasizing the critical need for heightened vigilance regarding Zika virus across India [1]. Despite the absence of reported Zika-associated microcephaly cases in India since 2016, state authorities are urged to maintain preparedness and swiftly report any suspected cases to the Integrated Disease Surveillance Programme (IDSP) and National Center for Vector Borne Diseases Control (NCVBDC) for a prompt response [2]. Over the past decade, sporadic outbreaks have highlighted its potential to spread rapidly, posing a growing concern for public health officials and communities alike [3].

India, as the world’s most populous country, faces unique challenges in disease management. The increased prevalence of vector-borne diseases, particularly those transmitted by Aedes mosquitoes, underscores the urgency of addressing the Zika virus [4]. India faces a critical public health concern due to the influx of international travelers from Zika-endemic regions. With the recent surge in Zika outbreaks within the country, the Zika virus is a growing major concern of public health in India [5]. ZIKV is an arthropod-borne virus belonging to the Flaviviridae family [6]. Zika virus was first identified in Uganda in 1947 when it was isolated from a sentinel rhesus monkey in the Zika forest [7]. The virus later spread to humans, with the first large outbreak occurring in 2007 on the Pacific Island of Yap in the Federated States of Micronesia [8]. Before this, only 14 cases of human Zika virus disease had been documented worldwide. Subsequent outbreaks occurred in French Polynesia (2013-2014) and South America (2015-2016), raising global concern [9].

In 2016, India reported its first confirmed Zika cases in Gujarat [10]. These cases were detected in Ahmedabad, marking the initial introduction of Zika virus into the country. The Krishnagiri district of Tamil Nadu reported a Zika virus case in July 2017 [11]. Maharashtra witnessed a recent surge in Zika cases, particularly in Pune. As of July 2, 2024, Maharashtra reported eight cases, of which six were in Pune, one in Kolhapur, and one in Sangamner [12]. Geographic information system (GIS) plays a pivotal role in tracking and analyzing the spread of Zika virus. By integrating data on weekly outbreaks from IDSP across India, GIS enables public health authorities to visualize hotspots, identify trends, and strategize targeted interventions to mitigate transmission [13].

Studying the characteristics of the Zika virus for the whole of India as well as region-wise is crucial for understanding transmission dynamics and time trends. Analyzing weekly outbreaks helps identify seasonal patterns, assess the effectiveness of control measures, and inform timely public health responses tailored to the region's unique epidemiological landscape [14]. Given the rapid geographic spread associated with recent ZIKV outbreaks, understanding the epidemiology and dynamics of the virus in different regions is crucial. India, with its diverse ecological landscapes and widespread mosquito populations, presents a unique setting for studying the transmission patterns and impact of ZIKV [15]. Furthermore, the potential introduction of more virulent strains from other endemic regions underscores the urgent need for comprehensive surveillance and preventive strategies tailored to local conditions [16]. This study was conducted with the rationale that by analyzing the geographical distribution and temporal trends of Zika virus outbreaks in India from January 2016 to December 2023 using GIS, policy implementation gaps can be identified, and targeted interventions can be developed to mitigate future public health crises. The primary research question guiding this study is as follows: What are the geographical distribution patterns and temporal trends of Zika virus outbreaks in India during this period? To address this question, the study set out to achieve several objectives: to analyze the geographical distribution of Zika virus outbreaks using GIS, to identify temporal trends in these outbreaks, and to highlight regions with significant concentrations of Zika virus cases and potential hotspots [17].

## Materials and methods

Study design

This study employed a secondary data analysis with descriptive and spatial analysis components to examine the geographic distribution and temporal trends of Zika virus outbreaks in India from January 2016 to December 2023. GIS and statistical methods were utilized to achieve these objectives.

Inclusion criteria

The study included data from all weeks between January 2016 and December 2023 where ZIKV outbreaks were reported and published on the IDSP website.

Exclusion criteria

Data from weeks where no ZIKV outbreaks were reported were excluded. Additionally, weeks with missing or inaccessible data due to technical issues on the IDSP website were excluded from the analysis.

Data collection

Data for the study were collected from the IDSP, which provides comprehensive surveillance data on various diseases, including the Zika virus. The dataset covered all reported Zika cases within the specified period. Due to occasional technical issues with the IDSP website, data for certain weeks were inaccessible and were therefore excluded from the analysis. Data collection involved downloading and compiling weekly reports from the IDSP portal, ensuring that all relevant information on Zika virus outbreaks was captured. The data were sourced from nationwide reports, ensuring representation across different regions of India.

Data analysis

Statistical analysis and GIS image generation were conducted using R software version 4.3.2 (R Foundation for Statistical Computing, Vienna, Austria). The analysis focused on identifying spatial and temporal patterns in Zika virus outbreaks across India. Heat maps and choropleth maps were created to visualize the geographic distribution of outbreaks, highlighting areas with significant concentrations of cases. Line diagrams were used to depict temporal trends, showing variations in outbreak occurrences over the study period. Descriptive statistics, including the interquartile range (IQR) and other relevant tests, were calculated to summarize the frequency and distribution of Zika virus cases. Spatial analysis techniques were employed to identify hotspots and patterns of disease spread.

Ethical considerations

The Institutional Ethics Committee (IEC) approval was not required for this study as it involved the analysis of publicly available data from the IDSP, maintained by the Government of India. The use of publicly accessible data ensured that no personal or sensitive information was accessed, maintaining the ethical integrity of the research.

## Results

The analysis of the IDSP data from 2016 to 2023 revealed a distinct seasonal pattern in Zika virus outbreaks in India. Outbreaks predominantly occurred during the post-monsoon season (47.62%), followed by the monsoon season (33.33%), and summer (19.05%). The chi-square test for season-wise distribution yielded a p-value of 0.28, indicating no statistically significant association between the season and the incidence of outbreaks. However, when combined, the monsoon with post-monsoon periods showed a significant association with a p-value of 0.0046 (p < 0.05). During the study period, only two deaths were reported, both in 2018 in Madhya Pradesh, involving an 18-year-old male with co-infection of dengue and Japanese encephalitis (JE) and a 23-year-old woman who succumbed to multi-organ failure.

Table 1 shows that the median duration between the onset and reporting of Zika virus outbreaks in India from 2016 to 2023 varied significantly based on settlement type. Rural areas had a median duration of five days (IQR: 4-8) compared to urban areas with two days (IQR: 0-3.5), with a significant p-value of 0.0373. For seasons, the median duration during the monsoon was 3.5 days (IQR: 1-6) and five days (IQR: 3-9) in summer, with no significant difference (p-value: 0.3746). First-time outbreaks had a median of four days (IQR: 1-6) versus three days (IQR: 5-13.5) for repeat outbreaks (p-value: 0.8642). Comparing years, the median duration for 2017-2021 was four days (IQR: 1-5), and for 2022-2023, it was 3.5 days (IQR: 2-7.5), with no significant difference (p-value: 0.6629).

**Table 1:** Analysis of the median duration between onset and reporting of Zika virus outbreaks in India (2016–2023). n = number of outbreaks. The p-values were calculated using the Mann–Whitney U test. A p-value less than 0.05 is considered statistically significant (*).

Category	Group (n)	The median duration between onset and reporting of the outbreak in days (IQR)	Mann-Whitney U value	p-value
Settlement	Rural (9)	5 (4-8)	18.5	0.0373*
	Urban (8)	2 (0-3.5)		
Seasons	Monsoon (14)	3.5 (1-6)	20.0	0.3746
	Summer (3)	5 (3-9)		
First or repeat outbreak	First (13)	4 (1-6)	25.5	0.8642
	Repeat (4)	3 (5-13.5)		
Years	2017-2021 (9)	4 (1-5)	27.0	0.6629
	2022-2023 (8)	3.5 92-7.5)		

**Figure 1:**
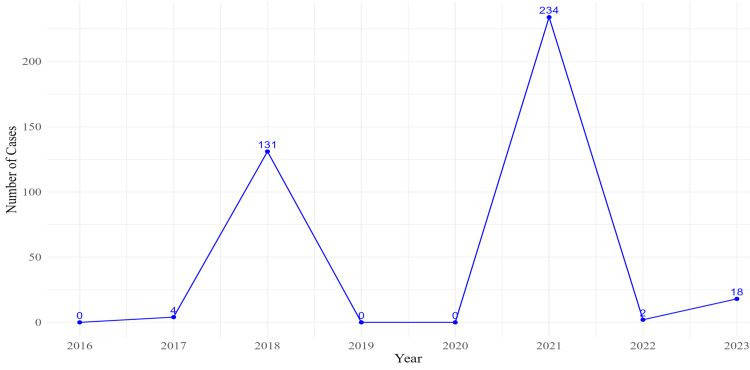
Trend of Zika virus cases in India from 2016 to 2023.

Figure 1 depicts the trend of Zika virus cases in India from 2016 to 2023. During the years 2018 and 2021, there were significant spikes, with reported cases of 131 and 234, respectively. There were no cases reported in 2019 and 2020. In 2022, only two cases were observed, followed by a rise to 18 cases in 2023.

**Figure 2:**
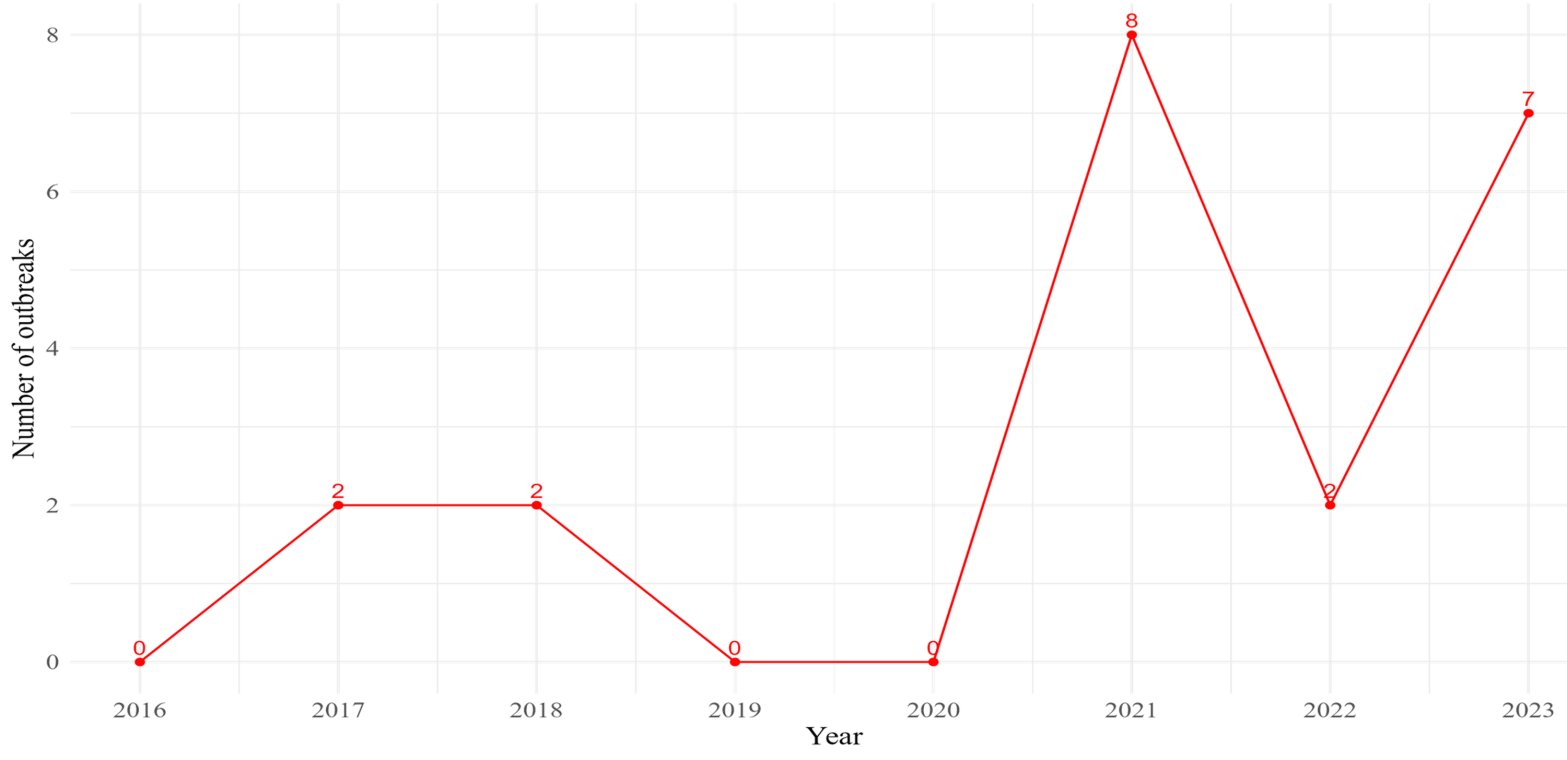
Trend of Zika virus outbreaks in India from 2016 to 2023.

Figure 2 shows the trend of Zika virus cases in India from 2016 to 2023. Notable outbreaks were recorded in 2017 and 2018, each with two outbreaks. The most significant spike in outbreaks was observed in 2021, with eight reported outbreaks. The years 2019 and 2020 saw no outbreaks. There were minor increases in 2022 and 2023, with two and seven outbreaks, respectively.

**Figure 3:**
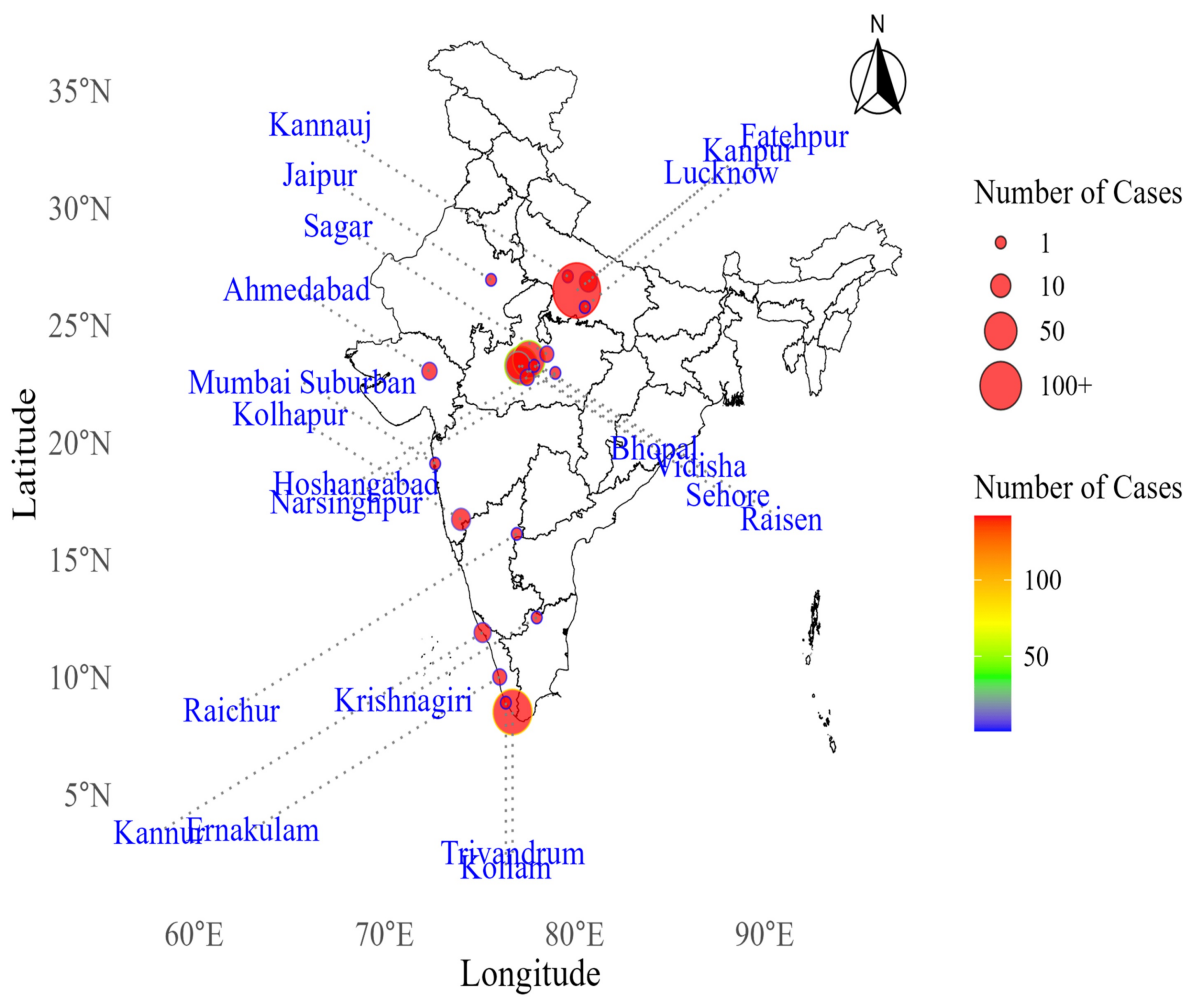
Heat map of Zika virus cases in India from 2016 to 2023. Image credits: Dinesh Asokan, Siva Prasad Reddy Bommu, Anjali Mall, and Geeta Pardeshi.

Figure 3 provides a heat map of the geographic distribution of Zika virus cases across India. The map highlights several hotspots in Uttar Pradesh, specifically in Kanpur, Fatehpur, and Lucknow. In Madhya Pradesh, significant concentrations are seen in Bhopal, Vidhisha, Sehore, Sagar, Hoshangabad, Raisen, and Narsinghpur. In Kerala, notable hotspots are observed in Trivandrum, Ernakulam, Kannur, and Kollam. In Maharashtra, cases occurred sporadically in Mumbai Suburban, Kolhapur, and Raigad, without significant concentrations. The color gradient and bubble sizes indicate the intensity of cases, with larger bubbles and darker colors representing higher case numbers.

**Figure 4:**
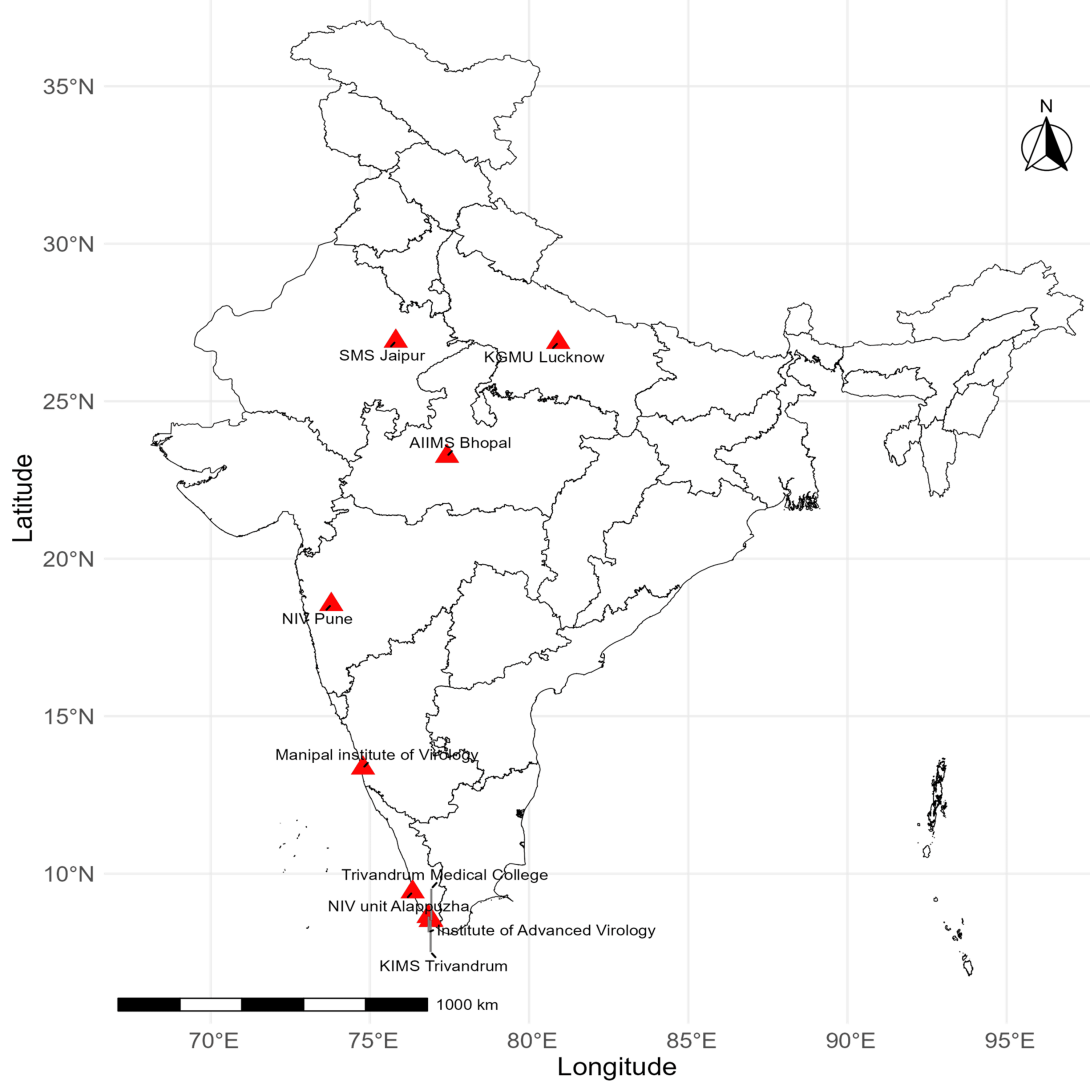
Choropleth map of Zika virus testing centers utilized in India from 2016 to 2023. NIV: National Institute of Virology; SMS: Sawai Man Singh Medical College; AIIMS: All India Institutes of Medical Sciences; KGMU: King George's Medical University. Image credits: Dinesh Asokan, Siva Prasad Reddy Bommu, Anjali Mall, and Geeta Pardeshi.

Figure 4 shows the locations of Zika research and testing centers across India. These centers include the National Institute of Virology (NIV) Pune in Maharashtra, Sawai Man Singh Medical College (SMS) Jaipur in Rajasthan, All India Institutes of Medical Sciences (AIIMS) Bhopal in Madhya Pradesh, King George's Medical University (KGMU) Lucknow in Uttar Pradesh, Manipal Institute of Virology in Karnataka, and several centers in Kerala, including National Institute of Virology (NIV) Unit Alappuzha, KIMS Trivandrum, Trivandrum Medical College, and the Institute of Advanced Virology. These centers are strategically located in regions with higher incidences of Zika cases to facilitate rapid response and research activities. These labs were crucial in the outbreak investigations from 2016 to 2023.

## Discussion

Our analysis of the IDSP data from 2016 to 2023 revealed a distinct seasonal pattern in Zika virus outbreaks in India, predominantly occurring during the post-monsoon season (10, 47.62%), followed by the monsoon season (7, 33.33%), and summer (4, 19.05%). Despite the lack of statistical significance in season-wise distribution (p-value = 0.28), the combined monsoon/post-monsoon periods showed a significant association (p-value = 0.0046), suggesting favorable conditions for mosquito breeding during these times.

The analysis reveals significant disparities in the median duration between the onset and reporting of Zika virus outbreaks in India, notably longer in rural areas (five days) compared to urban areas (two days), with a significant p-value of 0.0373. This suggests that rural regions face challenges such as limited healthcare infrastructure and delayed access to diagnostic facilities, leading to slower outbreak reporting. No significant differences were found based on season, outbreak frequency, or time period, indicating consistent public health responses across these variables. These findings highlight the urgent need for targeted interventions to improve outbreak reporting efficiency in rural areas and the continuous enhancement of public health strategies.

The geographic distribution of Zika virus cases across India, with hotspots in Madhya Pradesh, Uttar Pradesh, and Kerala, underscores the need for region-specific strategies to manage and control outbreaks. The presence of research and testing centers in these high-incidence regions, such as NIV Pune, SMS Jaipur, AIIMS Bhopal, KGMU Lucknow, and various centers in Kerala, has been crucial in facilitating rapid response and research activities. However, the observed gaps in outbreak reporting and the significant spikes in cases during 2018 and 2021 may be due to the availability of testing centers in these states, while other states lacking such facilities might be underreporting or not reporting cases at all. To address this issue, it is imperative to increase the number of ZIKV testing centers across the country to ensure comprehensive and timely diagnosis and reporting. This will help in promptly identifying and containing outbreaks.

The two deaths reported during the study period, both occurring in 2018, emphasize the potential severity of Zika virus infections, particularly when co-infections with other diseases such as dengue and Japanese encephalitis are present. This finding underscores the importance of integrated disease management strategies and the need for healthcare systems to be prepared for multi-faceted public health challenges. Moving forward, it is imperative to focus on strengthening diagnostic capabilities, enhancing surveillance systems, and promoting timely reporting to effectively manage and control Zika virus outbreaks in India.

The findings from this study align well with the broader understanding of Zika virus outbreaks both in India and globally. Previous research has consistently demonstrated the influence of seasonal patterns on the incidence of Zika virus. For instance, Bhardwaj et al. (2017) highlighted that the monsoon season creates favorable conditions for the breeding of Aedes mosquitoes, the primary vector for Zika virus transmission [18]. Dhimal et al. (2018) emphasized the importance of GIS in tracking arboviral diseases across Asia, which aligns with our study's use of GIS to map Zika virus outbreaks across India [19].

Biswas et al. (2020) provided a detailed analysis of the Zika virus outbreak in India in 2018, underscoring the significance of a rapid response and containment strategies, which is consistent with our findings of concentrated outbreaks in certain regions and the need for localized interventions [20]. Yadav et al. (2022) discussed challenges during the Zika outbreak in newer states of India, highlighting the critical need for expanding diagnostic capabilities to ensure timely detection and response [21]. This is particularly relevant to our findings, as limited testing facilities in the country may have contributed to the underreporting of Zika cases in regions distant from major testing centers.

Limitations

The study's reliance on IDSP government surveillance data is comprehensive but may have reporting gaps due to technical glitches and the COVID-19 pandemic, leading to incomplete outbreak data for certain weeks and no reported cases in 2019-2020. Additionally, focusing on specific outbreaks and regional responses may limit the generalizability of findings to other regions with different ecological and epidemiological contexts.

## Conclusions

Continuous surveillance and targeted interventions, especially during high-risk seasons, are crucial for managing Zika outbreaks in India. This study identified key geographical hotspots and temporal patterns through mapping and trend analysis, facilitating targeted interventions and more efficient resource allocation. Expanding testing capabilities, particularly in identified hotspots, and improving surveillance infrastructure are essential to capture a more accurate epidemiological picture and enhance response strategies. The study successfully fulfilled its objectives by comprehensively understanding the spatial distribution and temporal trends of ZIKV outbreaks, thereby effectively answering the research question.

To improve the management of Zika outbreaks, it is recommended that a dedicated percentage of the public health budget be allocated to enhancing existing laboratories at medical colleges or district head hospitals across all states for sample collection and storage. This should be complemented by investments in human resources and the training of healthcare workers for early diagnosis and response. Samples can then be transported to ZIKV testing labs for further investigations, providing a clearer picture of the geographical spread of the disease and enabling better preventive techniques specific to each region. Public awareness campaigns should also be implemented to encourage early symptom reporting and foster community involvement in outbreak prevention. By implementing these recommendations, India can improve its preparedness and response to Zika virus outbreaks, ultimately reducing the impact of the disease on public health.
